# Leukemic retinopathy, the first expression in a case of chronic myelomonocytic leukemia - a case report

**DOI:** 10.22336/rjo.2020.65

**Published:** 2020

**Authors:** Marina Istrate, Andreea Ciubotaru, Mihai Hasbei-Popa, Ana Maria Boariu, Daniela Adriana Iliescu

**Affiliations:** * Infosan Ophthalmology Clinic, Bucharest, Romania; ** “Victor Babeș” University of Medicine and Pharmacy, Timișoara, Romania; *** “Iuliu Hațieganu” University of Medicine and Pharmacy, Cluj-Napoca, Romania; **** “Carol Davila” University of Medicine and Pharmacy, Department of Physiology II, Bucharest, Romania

**Keywords:** Leukemic retinopathy, myelomonocytic leukemia, retinal hemorrhages, leukemic infiltrates

## Abstract

A 68-year-old male addressed to our clinic complaining of gradual loss of visual acuity and perceptual distortions. He had a history of extrathoracic hematoma and essential hypertension. The clinical assessment revealed bilateral retinal hemorrhages and white-green foveal and extrafoveal areas. The complete blood count (CBC) suggested a hematologic disorder.

## Introduction

Chronic myelomonocytic leukemia (CMML) is a rare clonal hematopoietic stem cell disease, characterized by an absolute monocytosis in the peripheral blood and tendency to develop splenomegaly, hepatomegaly and multiple serous effusions. The most reported chromosomal anomalies in patients with CMML are trisomy 8, complex karyotype, and abnormalities of chromosome 7 [**1**,**2**].

## Case report

A 68-year-old male presented for ophthalmological assessment complaining of gradual visual impairment and metamorphopsia. Anamnesis established he was diagnosticated with extrathoracic hematoma. The patient mentioned dizziness, tiredness and loss of appetite. 

Clinical eye exam showed:

• RE BCVA = 20/ 50

• LE BCVA = 20/ 40

• RE IOP = 13 mmHg

• LE IOP = 12 mmHg

• Normal ocular motility

**Fig. 1:**
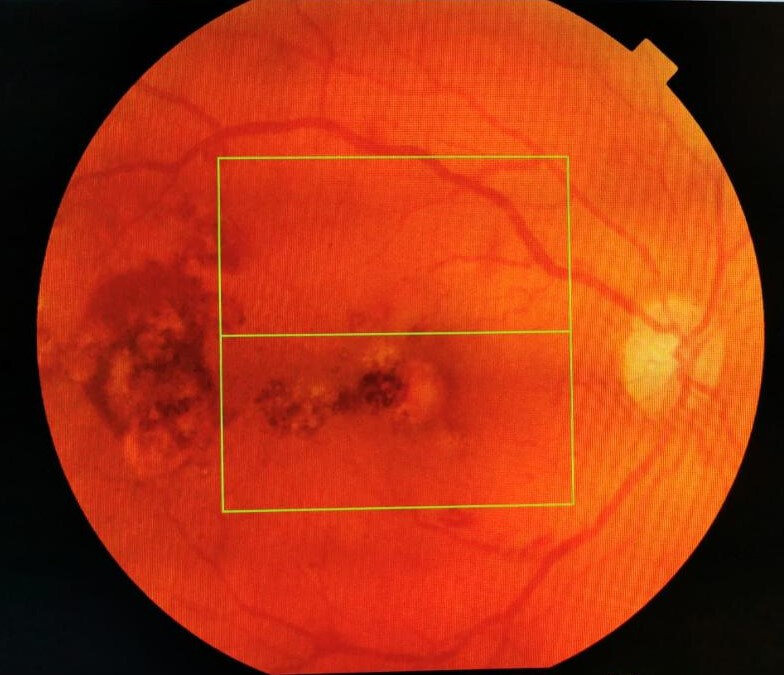
Posterior pole RE

**Fig. 2:**
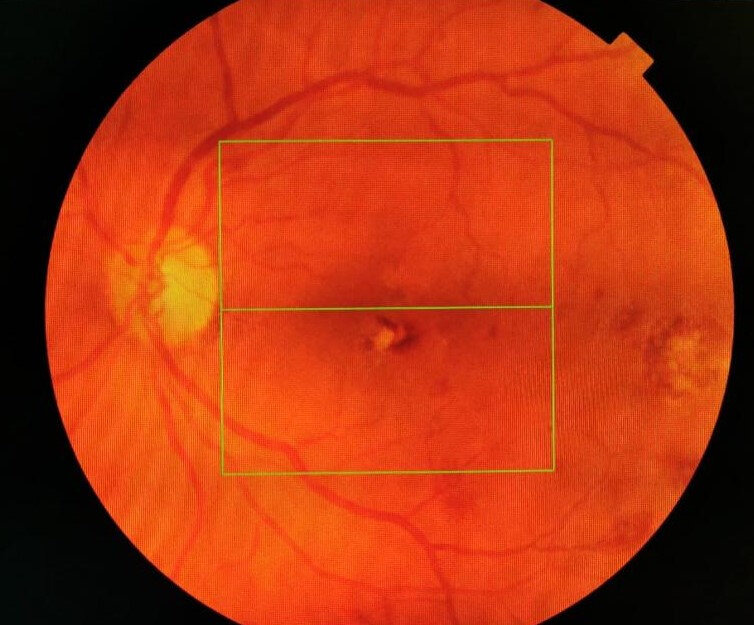
Posterior pole LE

Slit lamp examination of the anterior segment found moderate corticonuclear lens opacities, without other pathological changes. Fundoscopic examination showed multiple dot and blot retinal hemorrhages, irregular white-green foveal and extrafoveal round areas in both eyes, dilation and tortuosity of the retinal veins (**Fig. 1**,**2**).

**Fig. 3:**
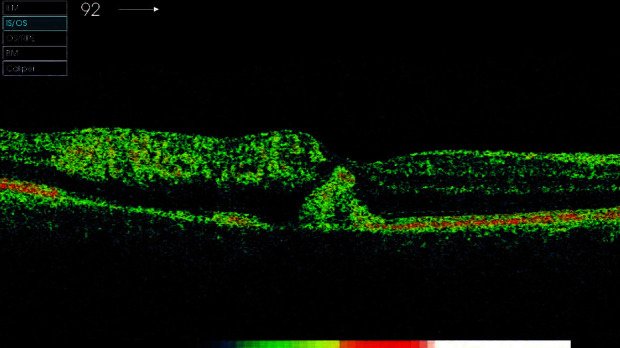
OCT image of RE

**Fig. 4:**
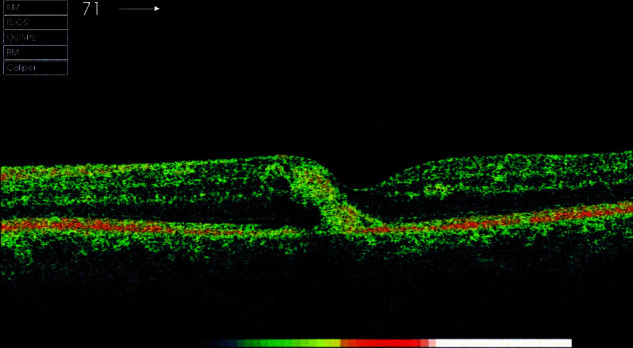
OCT image of LE

Optical coherence tomography revealed foveal hyperreflective lesions in both eyes (leukemic infiltrates) and subretinal fluid (**Fig. 3**,**4**).

Regarding the clinical aspects of this case, especially the appearance of retinal hemorrhages in the right eye, we recommended a hematological evaluation. Therefore, the complete blood count and biochemistry showed:

• Leukocytosis - 623320/ μl (normal range 4000-10000/ μl;

• Monocytosis - 37570/ μl (normal range 300-1000/ μl);

• Anemia, hemoglobin - 7300 mg/ dl (normal range 12600-17400 mg/ dl);

• PCR - 0,732 mg/ dl (normal range < 0.5 mg/ dl);

• LDH - 902 U/ L (normal range 135-225 U/ L);

• Thrombocytopenia - 96000/ μl (normal range 150000-450000/ μl);

• Elevated levels of immunoglobulins (IgA; IgG);

• Moderate macrocytic anemia;

• WBC left shift;

• Blood smear: atypical cells (25%) - Medium-large cells with inverted nucleo-cytoplasmic ratio. Nuclear chromatin condensation, with one or more nucleoli visible, basophile and granular cytoplasm.

The patient was hospitalized to the hematology department to complete the investigations. Subsequently, the diagnostic of chronic myelomonocytic leukemia (CMML) was established.

## Discussions

Eye involvement in hematologic diseases can be due to vascular changes (Roth’s spots, intraretinal hemorrhages, retinal neovascularization) or leukemic infiltration of the eyeball tissues. In leukemic disease, the retinal tissue is affected more frequently than in other structures. The first manifestations (due to hemodynamic instability) are venous dilatation and tortuosity. Retinal haemorrhages may occur in all layers and may invade the vitreous. Roth spots are retinal haemorrhages with white centers. The whitish fraction is composed of coagulated fibrin, leukaemic cells or septic emboli. The haemorrhages and infiltrates are mostly found in the inner layers of the retina. Besides Roth spots, retinal involvement may be represented by dot and blot, flame shaped and preretinal haemorrhages. Peripheral retinal neovascularization is an uncommon characteristic of chronic myeloid leukaemia (CML) [**3**,**4**].

Other features

• Cranial nerve palsies,

• Orbital involvement,

• Iritis and pseudohypopyon,

• Conjunctival involvements (cork screw vessels, spontaneous subconjunctival haemorrhages),

• Sterile ring ulcers,

• Scleral and episcleral infiltration,

• Choroidal infiltration (“leopard skin” appearance),

• Leukaemic chorioretinal infiltration,

• Opportunistic infections [**3**,**4**].

**Case particularity**

**Fig. 5:**
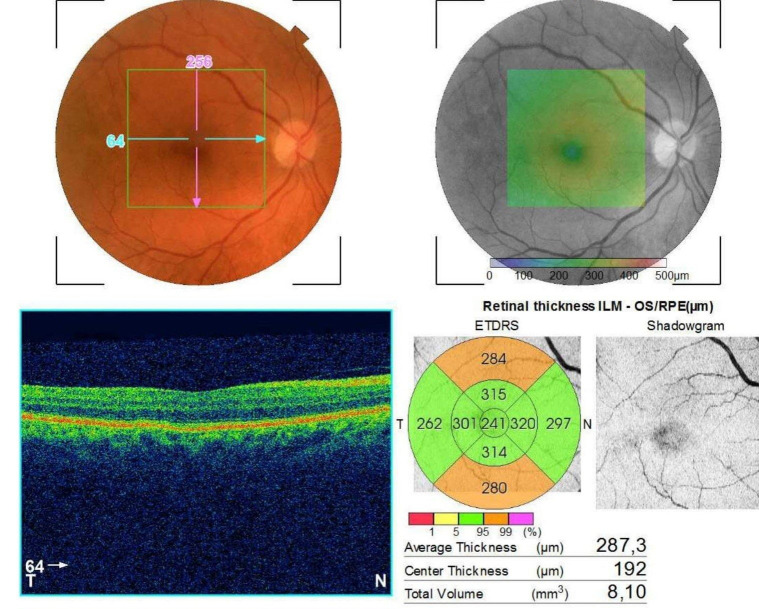
Posterior pole and OCT image of RE after treatment in the haematological department

**Fig. 6:**
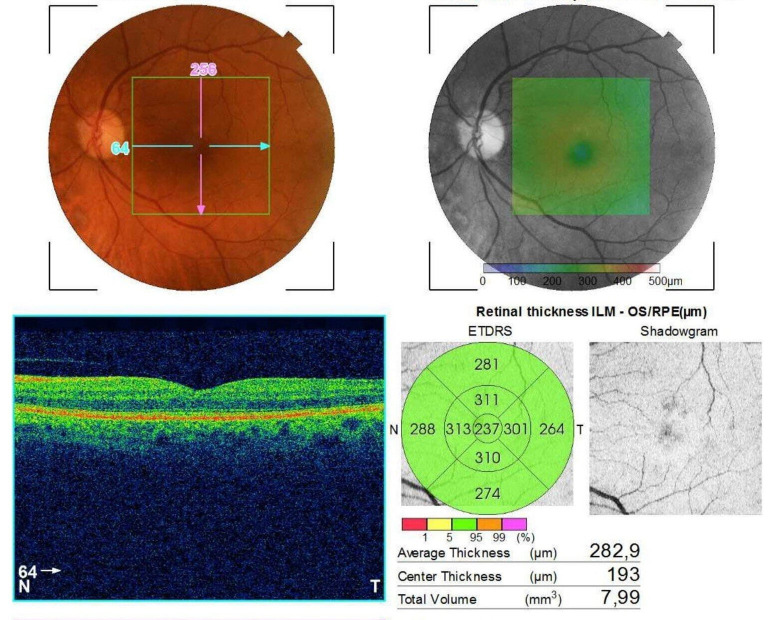
Posterior pole and OCT image of LE after treatment in the haematological department

Regarding the appearance of the posterior pole, optical coherence tomography and the patient’s age in this clinical case, the possibility to make a diagnosis confusion with neovascular age related macular degeneration occurred (**Fig. 5**,**6**).

**Treatment**

Standard chemotherapy using drug combinations can be a choice for young and old patients. Therapy with Hydroxyurea can help reduce monocyte count and lower the necessity for transfusions. Stem cell transplant (SCT) is the gold standard to heal young patients with chronic myelomonocytic leukemia (CMML). SCT could also be a therapeutic option for some older patients [**5**].

## Conclusion

The diagnosis of chronic myelomonocytic leukemia (CMML) can be intricate especially when the ophthalmic manifestation represents the first symptoms. The ophthalmologist has the role to recognize ocular manifestations in leukemic disorders and to perform clinical assessment with interdisciplinary collaboration.

**Conflict of interest**

The authors declare no conflict of interest.

**Informed consent**

Informed consent has been obtained from the individual included in this study.

**Authorization for the use of human subjects**

The research related to human use complies with all the relevant national regulations, institutional policies, is in accordance with the tenets of the Helsinki Declaration, and has been approved by the Ethics Committee of Infosan Ophthalmology Clinic.

**Acknowledgments**

None.

**Sources of Funding**

None.

**Disclosures**

None.
